# Persistent dyskinesias in patients with fetal tissue transplantation for Parkinson disease

**DOI:** 10.1038/s41531-021-00183-w

**Published:** 2021-04-23

**Authors:** Paul E. Greene, Stanley Fahn, David Eidelberg, Kimberly B. Bjugstad, Robert E. Breeze, Curt R. Freed

**Affiliations:** 1 Mt. Sinai School of Medicine, New York, NY USA; 2 Columbia University Medical Center, New York, NY USA; 3 North Shore University Medical Center, Manhasset, NY USA; 4 University of Colorado School of Medicine, Aurora, CO USA

**Keywords:** Parkinson's disease, Parkinson's disease

## Abstract

Cell transplants are being developed for patients with Parkinson disease (PD) who have insufficient benefit with standard medical treatment. We describe the clinical features of five patients who developed persistent dyskinesias after fetal dopaminergic tissue transplantation. All had levodopa-induced dyskinesias preoperatively. We implanted fetal mesencephalic dopaminergic tissue into the putamina bilaterally in 34 patients with advanced PD. They were not immunosuppressed. Five of 34 patients (15%) developed troublesome choreic or dystonic dyskinesias that persisted despite lowering or discontinuing medications. Attempts to treat the involuntary movements with amantadine, clozapine, anticholinergics, dopamine depletors and other medicines had limited success. Metyrosine eliminated dyskinesias but led to the parkinsonian “off” state. Increasing the dose of levodopa worsened the dyskinesias. Three patients required placement of pallidal stimulators, bilaterally in two and unilaterally in one patient who had only contralateral dyskinesias. The two with the bilateral stimulators had improvement in dyskinesias. The patient with the unilateral pallidal stimulator had a substantial reduction of the dyskinesias, but attempts to treat residual “off” symptoms with levodopa were limited by worsening dyskinesias. Although the number of patients developing these persistent dyskinesias was small, these five patients had dramatic improvement after transplant. As a group, they had milder Parkinson signs at baseline and improved to the point of having minimal parkinsonism, with reduction or elimination of levodopa therapy prior to developing persistent dyskinesias. These involuntary movements establish the principle that fetal dopaminergic tissue transplants can mimic the effects of levodopa, not only in reducing bradykinesia, but also in provoking dyskinesias.

## Introduction

Surgical treatments for advanced Parkinson disease (PD) are being developed for patients who fail conventional levodopa therapy because of severe “off” episodes and disabling dyskinetic “on” states. Open-label embryonic dopamine cell neurotransplantation has been performed by several groups with reports of lasting improvement in PD, and the procedure seems relatively safe compared to the reported benefit^1–14^. Controlled surgical trials have yielded mixed results^15,16^. Our double-blind study is the only controlled clinical trial to show significant transplant-induced improvement in objective measures of PD by clinical scoring^15^ and by timed testing^17^. Importantly, the transplants produced no change in cognitive performance^18^. Other studies reporting benefit have been uncontrolled. Pathologic findings in humans have demonstrated survival of neurons and functional connection with host brain^15,19–22^. In a few patients more than 10 years after transplant, a small percentage of implanted dopamine neurons have shown alpha-synuclein inclusions similar to Lewy bodies. The cause and physiologic importance of these inclusions is unknown^23–26^.

An increase of dyskinesias after transplant has been reported in some patients who had shown clinical improvement^1,5,7^, presumably caused by dopamine produced by the transplant adding to dopamine generated from levodopa. Such dyskinesias usually responded to reduction in levodopa doses^1,5^. A lessening of dopa-induced dyskinesias following transplantation has also been reported without reducing the dosage of levodopa^19^.

In January 1999, we completed the 12 month blinded phase of the first prospective, randomized placebo-controlled double-blind study of fetal tissue implantation for severe PD and subsequently reported those results^15,17,18,27–30^. As in prior studies, many patients developed increased dyskinesias. Subsequent reduction in levodopa doses improved this condition in most patients. However, in five subjects we observed that choreic and dystonic dyskinesias persisted despite the prolonged discontinuation of most or all levodopa. Following our report describing persistent dyskinesias^15^, Swedish investigators and their colleagues reevaluated their patients who had undergone transplantation and reported that some also had persistent dyskinesias^31^. The second controlled trial of human fetal tissue implantation for PD also observed dyskinesias in some patients in the “practically-defined off” state^16^, although not necessarily in the absence of levodopa. We now present the details, show videotape scenes of these persistent dyskinesias, and describe our attempts to deal with this problem using medications and deep brain stimulation.

## Results

### Patient histories

We present the patients in the temporal order that the persistent dyskinesias appeared.

Patient 122 (video segments 1–4) developed parkinsonism at about age 36 in 1984. His major problems were wearing-off with severe bradykinesia and rigidity while off and peak-dose levodopa-induced dyskinesias, especially in the face and neck.

He underwent fetal tissue implantation at age 47 in March 1996 during the double-blind phase of the trial. By one year after surgery, he improved substantially (see Table 1). During the first year after the implant, facial dyskinesias worsened but then improved after reducing drug doses. About 18 months after surgery, dyskinesias reappeared in the face, neck, and upper extremities and gradually became more severe than before surgery. In September 1997, he stopped all medications except for amantadine, but the dyskinesias persisted. Pergolide was restarted due to worsening of PD symptoms. His dyskinesias continued to worsen, and by 3 years after surgery, blepharospasm, torticollis and jaw-closing dystonia appeared.

**Table 1:** Quantitative results of the subjects with persistent dyskinesias.

	Patient	Patient	Patient	Patient	Patient
	122	116	131	104	133
Total UPDRS “off” at Baseline/1 year after transplant	52/11^a^	34/11^a^	54/24^a^	57/30^b^	41/29^b^
Total UPDRS “on” at Baseline/1 year after Transplant	11/10^a^	10/8^a^	18/7^a^	7/25^b^	9/4^b^
Motor UPDRS “off” at Baseline/1 year after transplant (UPDRS items 18–31)	34/7^a^	22/9^a^	33/12^a^	40/20^b^	30/21^b^
Motor UPDRS “on” at Baseline/1 year after transplant (UPDRS items 18–31)	8/7^a^	7/7^a^	11/3^a^	7/16^b^	6/3^b^
Dyskinesia scores (UPDRS items 32 and 33) at Baseline/1 year after transplant Item 32 (duration):	2.5/0^a^	1/1^a^	0.5/1^a^	4/4^b^	2/2^b^
Item 33 (disability):	3/0^a^	1/0^a^	0/0^a^	2/1^b^	0/0^b^
FDOPA PET score % improvement at 1 year after transplant	65%	68.5%	70.5%	17.5%	57.5%
Medication dose in levodopa equivalents Baseline/ 1 year after Transplant^c^	1150/200	1140/1215	500/400	700/425	480/125
Levodopa challenge: % improvement in motor UPDRS^d^	76.4%	68.2%	66.8%	76.9%	80.5%

FDOPA PET score = striatal to occipital ratio of FDOPA uptake, mean of left and right putamina.

Levodopa equivalents calculated according to the following ratios: 100 mg carbidopa/levodopa controlled release = 75 mg regular carbidopa/levodopa, 1 mg pergolide = 100 mg regular carbidopa/levodopa, 10 mg bromocriptine = 100 mg regular carbidopa/levodopa, 400 mg plain levodopa = 100 mg regular carbidopa/levodopa.

^a^Blinded UPDRS evaluations.

^b^UPDRS evaluations unblinded; Baseline is last evaluation prior to actual transplant, F/U UPDRS evaluation 1 year later.

^c^Medication doses given at baseline and 1 year after surgery for patients 116, 122, 131 and at last visit before actual transplant and 1 year after actual transplant for patients 104, 133.

^d^Dopa challenge at baseline for patients 116, 122, 131 and at last visit before actual transplant for patients 104, 133.

Over time, the facial dystonia improved, and he lowered his medications. As of September 2005, 9 years after implantation, he was taking no anti-Parkinson medication and was living independently. His daily walk increased from one to three miles per day. He preferred his transplanted state to preoperative because of the elimination of the severe “offs”. He continued to have severe, troublesome dysarthria, dysphagia, blepharospasm, oromandibular dystonia and head oscillations^29^. The abnormal head movements and dysarthria were partially relieved while walking.

Patient 116 (video segments 5–8) developed parkinsonism at about age 16 in 1977. He had a fetal tissue implant in January 1996 at age 35 during the double-blind phase of the trial. His major problem was wearing off, including “off” dystonia of the face, although he also had levodopa-induced dyskinesias. He did well for the first year after transplantation (see Table 1). About 18 months after implantation, he developed increasing dyskinesias. His dyskinesias improved after reductions in medications, but gradually worsened. By the end of the second year after implantation, he had periods of facial dystonia severe enough to interfere with speech. Controlled-release carbidopa/levodopa was stopped for 3 weeks, but dyskinesias and dystonia persisted. His remaining anti-Parkinson medication, pergolide, was stopped for the next 2 weeks, but his involuntary movements were only slightly better. For the next year, he took amantadine, trihexyphenidyl, cyproheptadine, clozapine and donepezil to tolerance without benefit. The tyrosine hydroxylase inhibitor, alpha-methylparatyrosine (metyrosine), controlled the dyskinesias but induced the “off” state, indicating that the transplant-induced dyskinesias are likely dopamine-mediated. He was taking only trihexyphenidyl 4–6 mg daily until January 2001, when he underwent placement of bilateral pallidal stimulators. There was modest improvement in facial dystonia after DBS. He continued to have troublesome dyskinesias, dystonia, and off periods, although these became milder over time. He rode a bicycle four to five miles per day, and his gait was nearly normal.

PATIENT 131. Videotape segments 9–12.

This patient developed parkinsonism at about age 45. Her main problem was deep “offs” particularly affecting gait, including off-freezing, although she also had generalized peak-dose levodopa-induced dyskinesias. Her response to levodopa was unpredictable, and she required a wheelchair when leaving the house because of unanticipated severe “offs.” She had been unable to undergo surgical repair of her left shoulder because of her surgeon’s concern that her severe dyskinesias would prevent wound healing.

She had a fetal tissue implant in October 1996 at age 56 during the double-blind phase of the study. For the first year after transplant, she did extremely well (see Table 1). On reduced doses of carbidopa/levodopa, her dyskinesias improved except at times of stress, and she was able to have elective shoulder surgery five months after implantation. Further reductions in levodopa combined with amantadine initially improved these dyskinesias, but the dyskinesias continued to worsen. By early 1999, she developed facial dystonia interfering with speech and troublesome leg dyskinesias. Parkinson symptoms returned on the left side. Pergolide 3.5 mg daily was replaced by ropinirole, and doses of medication fluctuated according to whether off’s or dyskinesias were more troublesome. Doses of ropinirole varied from 0.625 mg daily to 1.5 mg daily and doses of levodopa varied from pure levodopa without carbidopa 100 mg daily to carbidopa/levodopa 50/200 mg daily. Antiparkinson medications in low doses produced increased dyskinesias while improving her Parkinson “off” symptoms and mood. Metyrosine stopped her dyskinesias, but produced an “off” state as it had in patient #116. Baclofen, high dose amantadine, several dopamine agonizts, multiple antidepressants and benzodiazepines, gabapentin, donepezil and riluzole were not helpful.

In July 2000, she had placement of bilateral pallidal stimulators. By September 2000, her facial dystonia and limb dyskinesias were improved and were no longer troublesome. She added carbidopa/levodopa and metyrosine for her Parkinson symptoms and dyskinesias. She was admitted to hospital in August 2002 for falling. Her hospital course was complicated by sepsis, diffuse intravascular coagulation, hallucinations and agitation. She died of pneumonia in a rehabilitation facility 16 days after discharge. A brain autopsy revealed dopamine neurons in all transplant tracks with a total of 37,000 dopamine neurons surviving (detailed histologic analysis of brain innervation is being prepared for publication). The substantia nigra revealed dopamine neuron degeneration with Lewy bodies.

Patient 104 (video segments 13–16) developed parkinsonism at age 34 in 1986. His major problems were freezing while “off” and peak-dose, levodopa-induced dyskinesias, particularly of the right arm. He had a sham operation during the double-blind phase of the study and an open-label fetal tissue implant in April 1997 at age 45.

One year after the implant he had improved significantly (see Table 1). His gait and speech improved after transplantation. About 8 months after transplant, he developed dyskinesias, primarily in the right arm that responded to reductions in carbidopa/levodopa, but then recurred. He developed carpal tunnel syndrome of the right wrist requiring surgery. By September 1998 he was taking only carbidopa/levodopa 12.5/50 mg and pramipexole 2.25 mg daily. Amantadine improved but did not eliminate the dyskinesias. Addition of a single dose of metyrosine at night helped early morning right arm dyskinesias but produced an “off” state in the morning. To relieve the focal dyskinesias of the right arm, in December 2000 he underwent placement of a unilateral left pallidal stimulator. His dyskinesias were markedly improved by the stimulator.

Since October 2001, he has taken a single carbidopa/levodopa 25/100 in the morning. He has occasional troublesome dyskinesias of the left side when under stress. The patient considered himself much improved from his pre-transplant condition by the transplant-stimulator combination.

Patient 133 (video segments 17–19) developed parkinsonism at age 42 in 1986. His major problems were wearing-off episodes as well as levodopa-induced dyskinesias, particularly of the left arm. He had a sham operation during the double-blind phase of the study and an open-label fetal tissue implant in January 1999 at age 55. During the first year after the transplant, his “off” periods were milder (see Table 1).

After the transplant, dyskinesias increased, so levodopa was reduced. Controlled-release carbidopa/levodopa 25/100 mg was reduced to one per day and was discontinued by February 2000, 13 months after transplant. He continued to take trihexyphenidyl, selegiline, and amantadine. He was employed as an electrician. On September 11, 2001, he was working on the 34th floor of the first World Trade Center Tower attacked by a hijacked airplane. He walked down the 33 flights of stairs in the same 15 min as all others evacuating the building, ran five blocks to escape the dust cloud, and was not injured.

By October 2001, he indicated that left arm dyskinesias began at 9 to 10 a.m. and were bothersome about 1 h out of every four. Late in the afternoon, his right arm rather than his left might show intermittent dyskinesias.

Later in 2001, he experienced increasing “off” time, and restarted Sinemet which caused an increase in “on” dyskinesias. His levodopa-induced dyskinesias continued to fluctuate and were severe at times. Reduction of Sinemet and addition of ropinirole improved his fluctuations but did not eliminate them. In July 2002, 42 months after transplant, his UPDRS motor “off” score was 10 and “on” score was 5 with levodopa-induced dyskinesias present. Repeat FDOPA PET scan showed persistent putaminal fluorodopa uptake as we have found for at least four years in all subjects even without immunosuppression^32^. He continued to work full time as an electrician.

## Discussion

Patients selected for neurotransplantation had failed drug therapy primarily because of drug-induced dyskinesias or severe “off” episodes. All were candidates for some surgical intervention as an alternative treatment. In the first year after transplantation, many patients developed increased dyskinesias as in previous studies^1,5,7^, typically responding to a reduction in drug dosing.

Five out of 34 implanted patients (15%) developed persistent dystonia and/or choreic dyskinesias that were severe in four of the patients. The dyskinesias persisted in the absence of all anti-Parkinson medication in two patients and with minimal or no levodopa in the other three. Three (#122, #116, #131) of the five received the implant during the double-blind phase, and two (#104, #133) in the open-label phase. All five patients had substantial improvement in Parkinson symptoms in the first year after implantation with average improvement in UPDRS Total and Motor scores of 55.9 % and 56.4 %, respectively (see Table 1).

Because all persistent dyskinetic patients were in the younger group, these 19 younger transplanted patients were analyzed in more detail. It was important to identify those patients who improved from those who did not. “Improvement” was defined as a 10% or greater improvement in UPDRS Total “off” scores from baseline to 12 months after transplant. One patient was unable to return for the 12 month follow-up but was examined at 8 and 18 months. UPDRS scores at both time points for this patient exceeded the 10% improvement (44 and 33%, respectively), and the 18 month data were used for analysis for this patient only. Twelve of the 19 were improved while 7 were not. All five of the persistent dyskinetic patients were in the “improved” group.

**Fig. 1:**
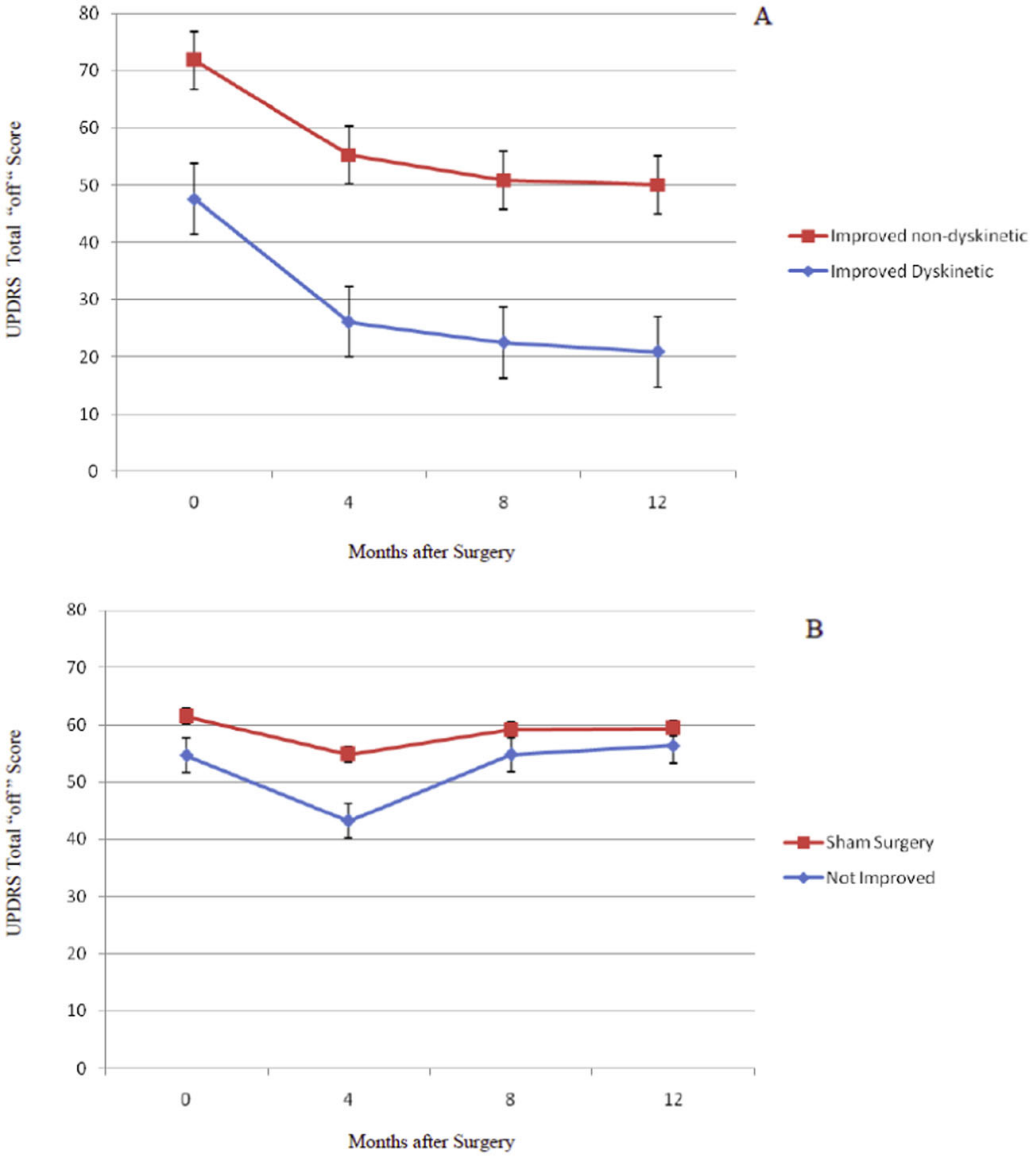
Change in Total UPDRS “off” scores for younger transplant and sham surgery patients. The graphs present the results only in the younger 21 subjects in the clinical trial because none of the older subjects developed persistent dyskinesias. This figure shows the Total UPDRS Off results from all implant operations that took place in either the double-blind phase (*N* = 10) or in the later open phase (*N* = 9) of the study. Analysis of the change in UPDRS total “off” scores 12 months after transplant surgery revealed that the 19 younger transplant subjects distributed into two groups, those who improved, *n* = 12, and those who did not, *n* = 7 (*p* < 0.05). The “Improved” group was defined as those with greater than 10% reduction in UPDRS Total “off” scores compared to baseline values. Panel **A** presents results from the younger subjects who improved after surgery. Five of those patients showed persistent dyskinesias (*n* = 5; 3 operated in double-blind phase and 2 in open phase), and seven were improved without dyskinesias (*n* = 7; 4 operated in double-blind phase and 3 in open phase). As can be appreciated by the parallel improvement lines, the magnitude of change in UPDRS Total scores was similar in the two groups (*p* > 0.05, n.s.). The dyskinetic patients differed from the non-dyskinetic subjects in that they had significantly milder Parkinson signs at baseline (*p* = 0.04) and improved to a level of minimal disease. The improved, non-dyskinetic subjects had more severe signs preoperatively and remained with moderate Parkinson “off” scores after 12 months. Panel **B** presents transplant patients who did not improve in the first 12 months after surgery (*n* = 7; 3 operated in double-blind phase and 4 in open phase) compared to sham surgery subjects (*n* = 11; all in double-blind phase). These groups were not different. All results are presented as mean ± SEM.

Figure 1, Panel A, presents the combined results of the double-blind and open surgeries with the five improved patients who developed dyskinesias and the seven improved patients who did not develop dyskinesias. While the two groups had similar improvement from baseline to 12 months in UPDRS Total “off” scores [t(9) = 2.22, *p* > 0.05, n.s.], the patients who became persistently dyskinetic after transplant had significantly milder disease at baseline [t(9) = 2.40, *p* = 0.04]. Their improvement at 12 months after transplant reached the point of minimal parkinsonian signs.

The persistent dyskinetic subjects were among the youngest in the study: three were under age 50 at the time of implantation, and two were 55 and 56 years old. In each case, the involuntary movements developed gradually and initially improved with reduction in anti-Parkinson medication. All had troublesome levodopa-induced dyskinesias prior to surgery, so that it was difficult to precisely date the transition from drug-induced to transplant-induced dyskinesias after surgery. Only after levodopa was discontinued or reduced to an extremely low dose (i.e., one-half tablet a day in Patient 104) could we be certain that we were dealing with persistent dyskinesias unrelated to levodopa dosing – a range of 13–24 months after surgery. Figure 1, Panel B shows that there was no change in the UPDRS Total “off” scores 12 months after surgery in either the sham surgery patients (*n* = 11) or the transplanted patients who did not improve after surgery (*n* = 7).

**Fig. 2:**
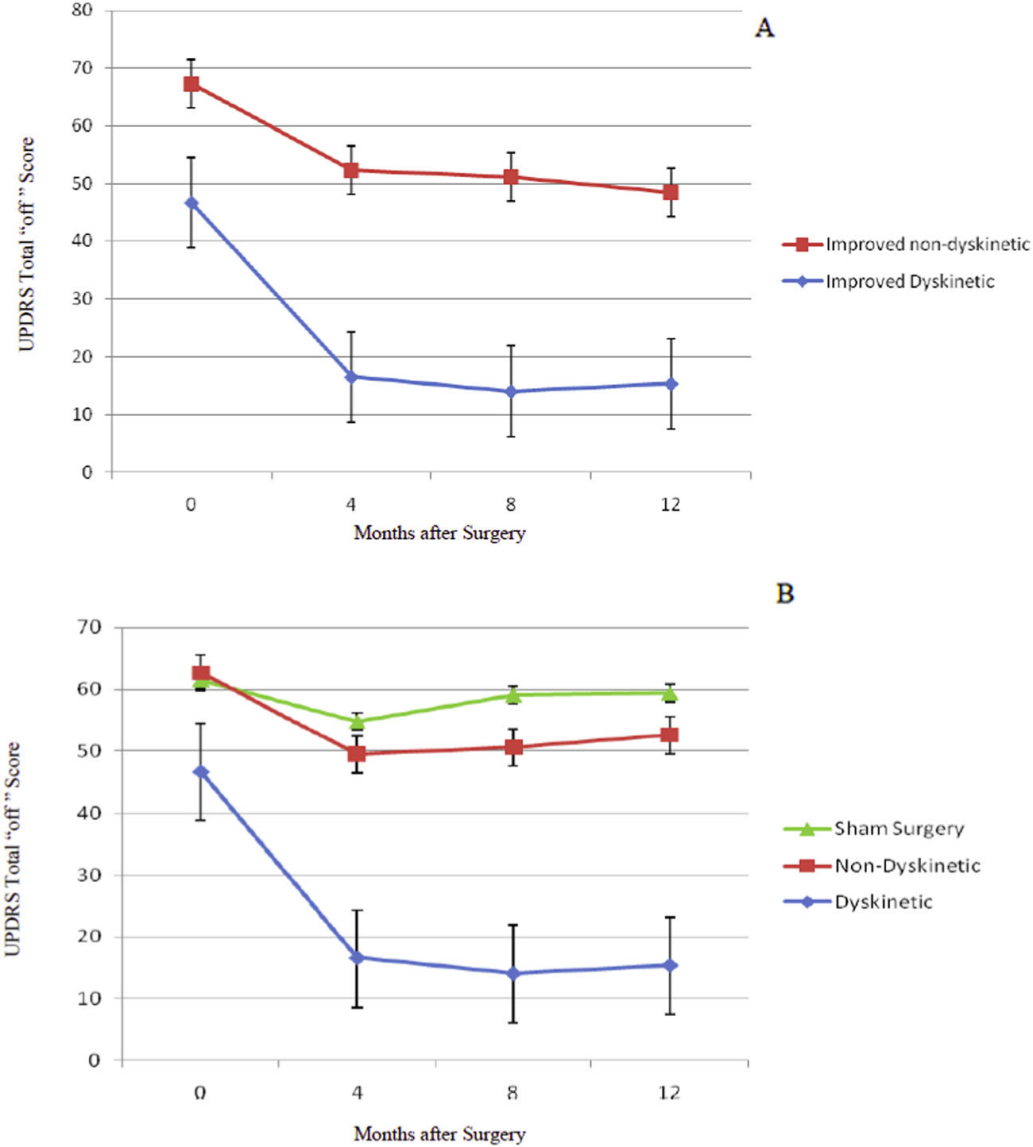
Change in total UPDRS “off” scores for younger transplant and sham surgery patients during the double-blind phase. This figure presents a subset of patients shown in Fig. 1. All data in this figure were obtained in the double-blind phase. Analysis of UPDRS Total “off” scores 12 months after surgery demonstrated that the 10 younger transplanted subjects distributed into two groups, those who improved, *n* = 7, and those who did not, *n* = 3. As in Fig. 1A, data are divided based on improvement, defined as greater than 10% reduction in UPDRS Total “off” scores compared to baseline. Panel **A** presents results from subjects who improved after surgery. Of the seven who improved, three showed persistent dyskinesias (Improved Dyskinetic, *n* = 3), and 4 were improved without dyskinesias (Improved non-dyskinetic, *n* = 4). As with the full group presented in Fig. 1A, the dyskinetic patients had milder Parkinson signs at baseline and improved to a level of minimal disease. The improved, non-dyskinetic subjects had more severe signs preoperatively and remained with moderate Parkinson scores after 12 months. Transplanted subjects who did not improve in the first 12 months after surgery (*n* = 3) had outcomes similar to the sham surgery subjects (*n* = 11) (data not shown). There were no placebo effects. Panel **B** presents results comparing the three dyskinetic subjects to all other transplanted patients, whether improved or not improved, and to sham surgery subjects. When presented using this format and analyzed by one-way ANOVA, the dyskinetic subjects were significantly improved compared to the combined Improved and Not Improved non-dyskinetic transplant patients and to the sham surgery subjects (*p* < 0.01) (Dyskinetic, *n* = 3; Non-Dyskinetic, *n* = 7; Sham Surgery, *n* = 11). There was no statistical difference between the Non-Dyskinetic and Sham Surgery groups in this small subset of patients.

Data restricted to the double-blind phase of the study were also analyzed with results similar to those obtained for all younger patients in the study as presented above and shown in Fig. 1. Of the 10 subjects in the younger group receiving transplants during the double-blind phase, 7 subjects improved and 3 did not improve. Figure 2A presents results from subjects who improved. Three developed persistent dyskinesias, and four did not. This subset of dyskinetic patients mirrored the larger group presented in Fig. 1; the dyskinetic patients had milder PD at baseline and at 12 months after transplant compared to subjects who improved without becoming dyskinetic. Subjects who did not improve were not different from the sham surgery group (data not shown). There were no net placebo effects. In Fig. 2, panel B, the 3 dyskinetic patients are compared to all 7 non-dyskinetic subjects, both improved and not improved. When analyzed by one-way ANOVA, the improved, dyskinetic subjects were significantly improved compared to sham surgery subjects and also compared to the combined non-dyskinetic transplanted groups (*p* < 0.01). There was no statistical difference between the Non-Dyskinetic and Sham Surgery groups. Sham surgery results in Figs. 1 and 2 are from the same 11 subjects evaluated during the double-blind phase.

The pattern of dyskinesias resembled those observed preoperatively, though there were also differences as noted above. Although we cannot determine for certain the pathophysiological basis of these involuntary movements, there are at least six pieces of evidence that dopamine produced by the transplants is the most likely cause:

1. As seen in the videotape segments, the *phenomenology* of the choreic and dystonic dyskinesias strongly resembles that of typical levodopa-induced dyskinesias.
2. *Anti-dopaminergic drugs*, such as tetrabenazine and metyrosine, reduced the severity of the dyskinesias. The drugs also brought back parkinsonian symptoms.
3. The five persistently dyskinetic patients had *statistically significantly greater fluorodopa uptake* by FDOPA PET than implanted patients without dyskinesias^29^, though in no case was uptake greater than seen in individuals without PD.
4. The five dyskinetic patients had greater *fluorodopa uptake* in the ventral and posterior left putamen on PET compared to the other 14 transplanted younger subjects who did not develop persistent dyskinesias^29^. This uneven pattern suggests local areas of higher levels of dopamine nerve terminals in these patients.
5. The *time course* of the dyskinesias was compatible with gradual outgrowth of graft neurites over months to years. The dyskinesias were delayed for up to 24 months after transplant. It is known that transplants continue to develop over a period of years^5,12,14^. This pattern also fits with the autopsy results seen in three subjects in this study. Subjects dying at 36 and 70 months after transplant had far greater distribution of dopamine neurites in the putamen than a patient dying at 7 months^15^.
6. Finally, there is a *strong correlation with clinical improvement*. Patients #116, #122 and #131, the three who were implanted in the double-blind phase, and patients #104 and #133 implanted during the open clinical trial, experienced among the most dramatic improvements in motor and total UPDRS “off” scores which resulted in minimal signs of Parkinson’s disease even as they went on to develop persistent dyskinesias. Their improved UPDRS scores were a strong contribution to the statistically significant improvement seen in the younger patient group (Figs. 1A and 2B). The combination of milder disease prior to transplant and improvement into the range of minimal Parkinsonism appeared to tip the dopamine balance toward dyskinesias in patients with a history of levodopa-induced dyskinesias.

While dyskinesias have been noted since the earliest reports of neurotransplantation (1, 5), these have usually responded to reductions in levodopa dose. There have been reports of increased dyskinesias after fetal tissue implantation in 12 patients^1,5,9,11,13^. In most of these patients, the dyskinesias resolved after a decrease in anti-Parkinson medications. Four of the 12 patients did not tolerate the decrease, and presumably the dyskinesias continued to be troublesome^21.13^. Olanow et al.^16,33^ described dyskinesias in the “practically-defined off” (about 12 h after the last dose of medicine), and most were mild in severity. One of their patients had more severe dyskinesias which responded to deep brain stimulation of the subthalamic nucleus^34^. These patients may not be comparable to ours since we do not know what would have happened to their dyskinesias if medications were tapered.

Although there were several technical differences between our study and other studies, the fact that persistent dyskinesias have been seen by others^12,13,16,31,33^ makes it unlikely that methodologic differences accounted for the dyskinesias seen in our subjects. Nevertheless, we list these differences in case any may have influenced transplant outcome. First, our series transplanted a total of 34 patients, more than any other group. This large number of subjects made it possible to see dyskinesias affecting 15% of subjects. We have been the only group that has used no immunosuppression. We implanted strands of solid tissue from embryos as opposed to cell suspensions. Our tissue was kept in culture for up to four weeks prior to implantation, and we placed tissue in the dorsal and ventral putamen with needles penetrating through the forehead. Most of the patients in our study were evaluated for *Parkin* mutations. One of the patients with persistent dyskinesias had compound heterozygous *Parkin* mutations as did two patients who benefited without persistent dyskinesias. Subjects were not screened for other Parkinson genes.

Because we were unable to alleviate these persistent involuntary movements with medications, we turned to surgery. We utilized deep brain stimulation of the internal globus pallidus for our patients with persistent dyskinesias, and this was partially effective. Other groups have treated persistent dyskinesias with either pallidal or subthalamic nucleus stimulation and reported reduction of dyskinesias^34–36^.

None of the older subjects (age > 60 years) developed these persistent dyskinesias. While older subjects had transplant survival and growth by ^18^F-fluorodopa PET scans that was equivalent to the younger subjects, none improved to the point of having minimal Parkinson signs^15,27^. By contrast, 5 of 19 (26%) of the younger transplant group developed dyskinesias. These patients experienced among the most dramatic improvements in total and motor UPDRS “off” scores, and their transplants replaced the need for levodopa. Because of the variability in transplant outcome, from no improvement to improvement that replaces the need for levodopa, there is a need to refine transplant methodology as well as patient selection.

What are the implications of persistent dyskinesias for the future of cell therapy? Because the transplants were able to replace the need for levodopa in some of our patients, the concept of transplantation as a potential therapy has been affirmed. As with levodopa therapy, cell transplants that produce maximal improvement in motor “off” symptoms also produce dyskinesias in susceptible patients. This quandary with levodopa therapy has been faced for more than four decades since its introduction, without resolution. Deep brain stimulation in our subjects can control dyskinesias. Ideally, either through patient selection or transplantation methodology, protocols could be developed to reduce the likelihood of dyskinesias.

We can speculate on ways that might avoid these persistent dyskinesias.

1. Studies in rodents^37^ and primates^38^ suggest that focal grafts induce dyskinesias whereas diffuse grafts do not. This is compatible with the focal “hot spots” in our dyskinetic subjects^30^.
2. Future transplant strategies could select patients who did not have disabling levodopa-induced dyskinesias pre-operatively. It may be appropriate to conduct a transplantation study earlier in the disease before levodopa-induced dyskinesias have appeared.
3. The site of transplantation in the striatum may be important. Because transplants into ventral putamen supply additional dopamine to areas responsible for control of head and arm movements^39^, and because the dopamine deficiency of PD is most marked in dorsal and posterior putamen^40^, it is possible that transplants restricted to dorsal putamen might reduce the likelihood of head and neck dyskinesias.
4. The availability of dopamine neurons differentiated from stem cells might reduce the variability in outcome seen with fetal dopamine cell transplants and reduce the likelihood of having no clinical improvement.

Whether dopamine replacement by cell therapy in the advanced PD patient is a worthwhile strategy has been questioned^41,42^. From our patients, it is clear that transplants replicate the effect of levodopa, including the tendency to produce dyskinesias in susceptible patients. Because transplants were able to provide persistent relief from “off” symptoms, four of the five persistently dyskinetic patients preferred transplants with or without DBS to their un-operated state. Unfortunately, the symptoms of PD that are intractable to dopamine replacement therapy, such as balance, cognitive function, and nonmotor symptoms, are also unaffected by cell transplantation or by deep brain stimulation of the subthalamic nucleus or internal pallidum. These intractable symptoms remain a challenging dilemma for the treatment of Parkinson disease.

Following the completion of our trial and the subsequent Olanow, et al., trial, in 2010 a European group (TRANSEURO) planned to transplant fetal mesencephalic dopamine neurons into 40 patients in a Phase I clinical trial^43^. A number of technical and administrative problems have prevented the completion of that trial. As of 2018, 11 patients have received transplants (Roger Barker, PhD, personal communication, March 2018).

For more than a decade, embryonic stem cells and induced pluripotent stem cells have been differentiated into dopamine neurons by recapitulating normal embryonic development of dopamine neurons. These stem cell-derived dopamine neurons are planned to be transplanted into Parkinson patients before 2020^44^.

Implants of stem cell-derived dopamine neurons into Parkinson patients are likely to improve bradykinesia and other levodopa-responsive symptoms, thereby replicating the effects of fetal dopamine cell transplantation. The hope is that a standardized cell source will produce less variability in clinical outcome. Our results indicate that patients who are selected should have an excellent preoperative response to levodopa (> 75% improvement in UPDRS motor scores without dyskinesias). These patients should improve up to the best effects produced by levodopa. If these outcomes are observed, then neurotransplantation may become an available and common treatment for advanced Parkinson’s disease.

## Methods

### Double-blind study

This double-blind, controlled fetal tissue transplant study was a multicenter effort involving the University of Colorado School of Medicine, Columbia University Medical Center, and North Shore University Hospital to determine whether implants of embryonic mesencephalic dopamine cells improve PD compared to a sham surgical procedure, with patients and examining physicians blinded to the treatment assignments. Forty patients with PD were enrolled into the study. Requirements were PD symptoms of at least seven years’ duration and disabling symptoms including dopa-induced dyskinesias and severe “off” episodes despite optimal drug treatment. Planned enrollment required half the subjects to be ≤60 years (labeled as younger) and half older, with a preplanned analysis between these groups. Collection and preparation of fetal tissue and the technique for implantation have been described in detail elsewhere^15^. The 12-month blinded phase was followed by an open phase during which sham surgery subjects could receive mesencephalic tissue implants if they chose to do so. The protocol and consent forms were approved by the IRB at the Columbia University Medical Center. The consent form was signed by all participants and included the potential for publication of videos in medical journals.

### Reporting summary

Further information on research design is available in the Nature Research Reporting Summary linked to this article.

## Supplementary information

Supplementary Movie 1

Reporting Summary

## Data Availability

Table 1 and Figs. 1 and 2 contain raw data. There is no restriction on availability of raw data which is available from CRF.
